# Mediating role of Interleukin-6 in the predictive association of diabetes with Hippocampus atrophy, Amyloid, Tau, and Neurofilament pathology at pre-clinical stages of diabetes-related cognitive impairment

**DOI:** 10.1016/j.bbih.2025.101031

**Published:** 2025-06-16

**Authors:** Asma Hallab, The Health and Aging Brain Study (HABS-HD) Study Team, HABS-HD MPIs, Sid E. O'Bryant, Kristine Yaffe, Arthur Toga, Robert Rissman, Leigh Johnson, the HABS-HD Investigators, Meredith Braskie, Kevin King, James R. Hall, Melissa Petersen, Raymond Palmer, Robert Barber, Yonggang Shi, Fan Zhang, Rajesh Nandy, Roderick McColl, David Mason, Bradley Christian, Nicole Phillips, Stephanie Large, Joe Lee, Badri Vardarajan, Monica Rivera Mindt, Amrita Cheema, Lisa Barnes, Mark Mapstone, Annie Cohen, Amy Kind, Ozioma Okonkwo, Raul Vintimilla, Zhengyang Zhou, Michael Donohue, Rema Raman, Matthew Borzage, Michelle Mielke, Beau Ances, Ganesh Babulal, Jorge Llibre-Guerra, Carl Hill, Rocky Vig

**Affiliations:** a Psychiatry Neuroimaging Laboratory - Psychiatry and Radiology Departments – Mass General Brigham, Harvard Medical School, Boston, MA, USA; b Biologie Intégrative et Physiologie (BIP) – Parcours Neurosciences. Faculté des Sciences et Ingénierie, Sorbonne Université, Paris, France; c Charité - Universitätsmedizin Berlin, Corporate member of Freie Universität Berlin and Humboldt-Universität zu Berlin. Berlin, Germany; d Pathologies Du Sommeil. Faculté de Médecine Pitié-Salpêtrière, Sorbonne Université, Paris, France

**Keywords:** Cognition, Insulin resistance, Aging, Brain, Cytokine, Neuroinflammation

## Abstract

**Introduction:**

Type-2 diabetes (T_2_DM) has been associated with higher dementia risks, but the mechanisms are still unclear, and there is increasing evidence of the role of cytokines. Interleukin-6 (IL-6) mediating effect has never been explored.

**Methods:**

The study included a subset of 1927 community-dwelling participants from the Health and Aging Brain Study: Healthy Disparities (HABS-HD) cohort with complete data. Cross-sectional and longitudinal analyses were performed. Associations were studied using multivariable linear, logistic, and mediation analysis with non-parametric bootstrapping.

**Results:**

T_2_DM and IL-6 were associated with worse executive function, Hippocampus atrophy, lower Aß_42_/Aß_40_ ratio, and higher Aß_40_, Aß_42_, total Tau, and NfL levels. IL-6 mediated 5 % of the association of T_2_DM with Aß_40_ ([1.5 %–10 %], *p-*value<2 × 10^−16^), 4 % with Aß_42_ ([0.7 %–11 %], *p-*value = 0.014), 8 % with TMT-B ([0.2 %–35 %], *p-*value = 0.046), 11 % with total Tau ([2.5 %–40 %], *p-*value = 0.010), 5 % with NfL ([1.6 %–8 %], *p-*value<2 × 10^−16^), and 12 % Hippocampus atrophy ([3 %–49 %], *p-*value = 0.004). The results, except TMT-B, were replicated in the longitudinal analysis of long-lasting T_2_DM on non-previously diagnosed cognitive impairment.

**Conclusions:**

The study captured a pre-clinical stage of the T_2_DM-dementia association. The mediating effect of IL-6 is a novelty that has to be further explored and accounted for in risk stratification and preventive measures, particularly in ethnic minorities.

## Graphical abstract

**Figure:**
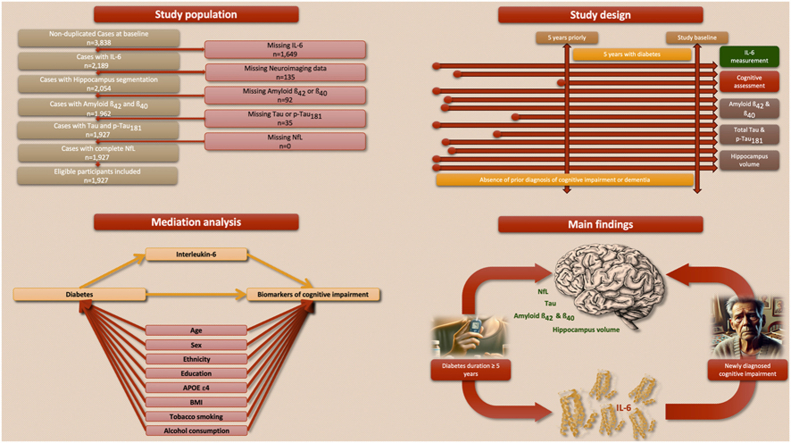

## 1 Introduction

Type 2 Diabetes Mellitus (T_2_DM) is one of the most geographically and demographically widespread non-communicable diseases (Zhou et al., 2024) and is recognized as a global public health challenge owing to its association with increased morbidity and mortality rates (Life expectancy associated with different, 2023). Despite treatment availability, recent studies have raised serious concerns about the rapidly rising incidence (van Sloten et al., 2024) and insufficient coverage of specialized medical care, particularly among people with disadvantaged backgrounds and in lower-resource settings (Zhou et al., 2024). The asymptomatic evolution of diabetic pathophysiology over the first years leads to its discovery at advanced stages when “classical” and non-classical complications have already evolved (Tomic et al., 2022). While some of them might be reversible with adherence to regular treatment and healthy lifestyles, others might cause severe organ-specific damage and chronic disabilities (Eid et al., 2019).

Cognitive disorders and dementia have also recorded increasing trends in the last decades, primarily owing to the growth and aging of the world population (Nichols et al., 2022), but also due to the changing lifestyles, and emerging obesity and glucose-related disorders in both industrialized and developing regions (Feng et al., 2025). It is estimated that around 153 million persons will be affected by dementia in 2050 (Nichols et al., 2022). Alzheimer's disease (AD) is characterized by Amyloid, Tau, and Neurofilament chain (NfL: a newer biomarker of neurodegeneration) pathology (ATN), which summarizes neural injury and accumulation of amyloid and tau plaques in the brain. It is the most prevalent dementia type worldwide, followed by vascular dementia (Kalaria et al., 2008). While each of these dementia types has a distinct pathophysiology (Wolters and Ikram, 2019), they generally tend to co-occur in older adults (Jørgensen et al., 2020).

T_2_DM demonstrates complex and multifaceted associations with neuropsychiatric and cognitive disorders (Fanelli et al., 2022; Geijselaers et al., 2015; Biessels et al., 2014). Microvascular complications are well-recognized mechanisms described in stroke (deep hemorrhagic stroke and lacunar ischemic stroke) and consequent dementia in patients with T_2_DM (van Sloten et al., 2020). Thus, the association of T_2_DM with non-vascular brain pathology (van Gils et al., 2024), low-grade inflammation (Bowker et al., 2020), and the multisystemic aspect of immunity-related complications (Gunes et al., 2023; Peters et al., 2016) implies the involvement of other actors that need to be better understood. Insulin resistance is a key mechanism in T_2_DM, causing impaired glucose metabolism in different organs, including the brain and, specifically, neurons, glia, and astrocytes, all of which are provided with insulin receptors (Scherer et al., 2021). Insulin receptors are also located in the blood-brain barrier, and their impairment is associated with AD pathology (Leclerc et al., 2023). On the other side, aging, social stress, overnutrition, disruption of the circadian rhythm, and sedentary lifestyles can trigger brain insulin resistance, which results in an increase in sympathetic nerve activity and reduces the activity of the Vagus nerve (Scherer et al., 2021). This leads, respectively, to lipotoxicity and hyperglycemia and, consequently, to systemic insulin resistance (Scherer et al., 2021). Brain insulin resistance is also associated with impaired body weight regulation and higher visceral fat distribution, increasing the risk of cardiometabolic complications (Kullmann et al., 2020; Heni, 2024). High plasma lipid and glucose levels are associated with elevated systemic pro-inflammatory cytokines and chronic inflammatory remodeling in various tissues (Daniele et al., 2014). This status of low-grade inflammation predicts the onset and evolution of several neuropsychiatric disorders (Palmer et al., 2024; Osimo et al., 2019; Kim et al., 2020). Low-grade inflammation might, therefore, play a significant role in diabetes-related neurodegeneration and cognitive decline. While the evidence of reciprocal brain-insulin-body crosstalk has been largely discussed in the last few years, the concomitant role of inflammation remains unclear.

Understanding the potential implications of systemic inflammation in the association between T_2_DM and cognitive impairment in middle-aged and older adults has promising potential to provide new pathways toward preventive and therapeutic options. However, most published studies explored simplistic associations in cross-sectional designs and were limited to two of those three variables in each of their study question. None explored the integrated role of Interleukin-6 (IL-6) in the diabetes-brain constellation, specifically whether there is a mediating effect of IL-6 on the path between diabetes and biomarkers of cognitive decline. Furthermore, previous studies were performed in middle-aged and predominantly White populations, and there is a lack of data on older adults and less-represented ethnic groups, those known for being exposed to higher risks of insulin resistance and cognitive decline (van Sloten et al., 2024; Mendenhall et al., 2017).

The first aim of this study was to assess the mediating effect of IL-6 in the association between T_2_DM and biomarkers of neurodegeneration and cognitive impairment. The second aim was to evaluate the mediating role of IL-6 in the longitudinal association between long-lasting T_2_DM and neurodegeneration-specific biomarkers in multiethnic community-dwelling middle-aged and older adults without previously diagnosed cognitive decline.

## 2 Methods

The study has been performed following the Strengthening the Reporting of Observational Studies in Epidemiology (STROBE) guidelines (von Elm et al., 2007).

### 2.1 Study population

The study population is a subset of the Health and Aging Brain Study – Healthy Disparity (HABS-HD) cohort. This NIH-funded study was performed at the Institute for Translational Research - 10.13039/100008973University of North Texas 10.13039/100018696Health Science Center as an extension of the HABLE study (O'Bryant et al., 2021). The first phase was initiated in 2017 with the general aim of studying health disparities in Mexican Americans compared to non-Hispanic White controls. From 2021, 1000 African American participants were enrolled in the cohort. HABS-HD is a community-based study; participants were recruited at several social events, and the study has been advertised in the media. Participants were encouraged to further advertise for the study, and snowball enrollment was commonly reported. Adults aged 50 and older were included, and those with type 1 diabetes, any form of dementia other than Alzheimer's type, severe mental illness (except depression and anxiety), alcohol and substance use disorders, active infection or cancer, and severe physical illnesses interfering with cognition (exp., end-stage chronic kidney disease, chronic heart failure) were not eligible (O'Bryant et al., 2021).

Participants underwent clinical, neuropsychiatric, biological, and neuroimaging investigations at baseline and a follow-up rhythm of 24–30 months intervals. However, many participants were lost to follow-up at 24 months, and, therefore, the current analysis is based on the baseline data of the 6th data release.

Procedures contributing to this work were all compliant with the Helsinki Declaration of 1975, as revised in 2013, and with the ethical standards of the relevant national and institutional committees on human experimentation. Ethical approval was obtained from the local institutional review board (University of North Texas Health Science Center). Written informed consent was obtained from every participant. The current research consists of a secondary analysis of anonymized data and was performed in accordance with the data use agreement (with Hallab), followed by an authorization for publication.

### 2.2 Exposure

The diagnosis of T_2_DM was based on self-disclosure of medical history of T_2_DM, fasting blood glucose (mg/dL) levels, glycated hemoglobin (HbA1c) (%), type of medication (self-disclosed), and date of onset (self-disclosed). As previously mentioned, type 1 diabetes was an exclusion criterion from the source cohort. No clinical recorders were consulted in this community-based study. In T_2_DM participants, further relevant measures were reported: fasting serum Glucose levels (mg/dL), age of onset of T_2_DM, type of treatment, healthy diet, and regular physical activity.

### 2.3 Outcomes

#### 2.3.1 Cognitive impairment

The main outcome of the study was cognitive impairment as a binary variable at baseline. “No cognitive impairment” was the reference negative value (Ref = 0). The presence of mild cognitive impairment (MCI) or dementia was labeled as “cognitive impairment” and was accorded a positive value (positive = 1). Questions on past medical history were analyzed to identify cases with no priorly diagnosed cognitive impairment or dementia (any etiology).

Furthermore, results of the mini-mental status examination (MMSE) total score (in points) (Folstein et al., 1975) and trail-making-test B (TMT-B) (in seconds) (Reitan, 1958) were evaluated.

#### 2.3.2 Amyloid β_42_ and β_40_, total tau, phosphorylated Tau_181_, and NfL

Amyloid β_42_ (Aß_42_) and β_40_ (Aß_40_) levels were assessed in plasma (pg/mL). A ratio was calculated based on the value of Aß_42_ on Aß_40_ levels. Similarly, total Tau, phosphorylated Tau 181 (p-Tau_181_), and Neurofilament Chain (NfL) levels were measured in plasma (pg/mL). To ensure the homogeneity of the findings, only values that were assessed during the same phase using the Quanterix ultra-sensitive Simoa® (Single Molecule Array) technology were considered.

#### 2.3.3 Hippocampus volume

All participants underwent 3 T magnetic resonance imaging (MRI) at the same site (two RMI scanners were used: MAGNETOM Skyra and MAGNETOM Vida 1, Siemens Healthineers®) and based on the ADNI 3 protocol (Gunter et al., 2017). Deep-learning T1-based segmentation was performed using Hippodeep (Thyreau et al., 2018). The Hippocampus volume was calculated as the sum of the right and left Hippocampi (mm^3^). Hippodeep-measured intracranial volume (ICV) (mm^3^) was reported.

### 2.4 Mediator

IL-6 was measured in the fasting blood plasma at baseline (pg/mL). It was assessed using the Quanterix ultra-sensitive Simoa® at the same study phase with plasma ATN measurements.

### 2.5 Confounders

Age (years), sex (females vs. males), ethnicity (self-disclosed “non-Hispanic White”, “Hispanic”, and “Black”), educational level (years), Apolipoprotein E (APOE) ε4 positivity (negative when none, and positive when one or two ε4 alleles are present), current tobacco smoking (binary), alcohol consumption (binary), and body-mass index (BMI); calculated as weight (Kg)/Height (m)^2^.

Models where Hippocampus volume was included as the dependent variable were additionally adjusted to the intracranial volume (ICV) (mm^3^) and MRI Scanner. Models where Aß_40_, Aß_42_, their ratio, total Tau, p-Tau_181_, and NfL were included as dependent variables were additionally adjusted to the estimated glomerular filtration rate as a continuous variable (mL/min/1.73 m^2^), based on the race-free CKD-EPI_2021_ equation (Miller et al., 2021).

### 2.6 Inclusion criteria

Only cases with complete data on IL-6, Aß_40_, Aß_42_, total Tau, p-Tau_181_, NfL, and total Hippocampus volume were included. All cases had fully available information on diabetes and cognition-related diagnoses. Cases missing the MMSE total score or TMT-B duration were eligible.

### 2.7 Statistical analysis

The statistical analysis was performed with RStudio version 2024.12.1 (Posit Software®, PBC, USA). For the selection of relevant outcomes, false-discovery rate (FDR) adjusted *p-*values were calculated to reduce the risk of Type I error. The significance level of the two-sided *p-* or *p*_*FDR*_*-*values was set at 0.05.

#### 2.7.1 Dealing with missing values in covariates

Covariates’ values missing completely at random were counted and reported as proportions (%). For missing proportions <1 %, continuous variables were imputed by the corresponding median value, and count variables by the null value.

#### 2.7.2 Exploring the normality of the distribution

Raw variables and their log-transformed values were plotted to evaluate the skewness in the data. The Shapiro-Wilk test was performed for each. Largely skewed variables were dichotomized into clinically relevant categories.

#### 2.7.3 Data description and group comparison

Percentages (%) and median with interquartile range (IQR) were used to present data. Groups were compared using the Wilcoxon rank sum test or Kruskal-Wallis rank sum test for continuous variables and Pearson's *Chi*-squared test (including Fisher's exact test) for count variables. Associations were explored using adjusted linear or logistic regression. Interaction terms were introduced when indicated.

#### 2.7.4 Cross-sectional analysis

Confounder-adjusted regression analysis was based on T_2_DM or IL-6 levels (pg/mL) as an independent variable, cognition-specific biomarkers as binary (cognitive impairment, MMSE-impairment, TMT-B impairment), or as continuous dependent variables (plasma Aß_40_ & Aß_42_ levels (pg/mL), their corresponding ratio (Aß_42_/Aß_40_), Tau and p-Tau_181_ levels (pg/mL), NfL (pg/mL), and total Hippocampus volume (mm^3^)). Only associations with *p*_*FDR*_-value <0.05 (in both T_2_DM and IL-6 corresponding models) were eligible for mediation analysis.

#### 2.7.5 Mediation analysis

Mediation analysis with 1000-fold non-parametric bootstrapping of 95 % CI based on the percentile method was applied. The same confounders were introduced in the mediator and outcome models. Hippocampus-specific mediation analysis was additionally adjusted for ICV (mm^3^) and MRI scanner. Plasma ATN-specific mediation analysis was additionally adjusted for eGFR value (mL/min/1.73 m^2^). The estimates of the mediated effect (ACME), direct effect (ADE), and total effect were visualized with their corresponding 95 % CI. Rounding to the second decimal was applied, except for variables with very low effect values. The proportion mediated was calculated as the value of the mediated effect from the total effect and reported in a fully rounded percentage (for an easier interpretation of the data) with the corresponding 95 % CI and *p-*value.

#### 2.7.6 Longitudinal analysis

To understand the underlying pathophysiology in a longitudinal framework, a second analysis was performed. After excluding cases with prior cognitive impairment or dementia diagnosis and those with recently diagnosed diabetes (within the last five years), mediation analysis was based on long-lasting T_2_DM (at least for five years) as exposure, FDR-adjusted cognition-specific biomarkers as an outcome (baseline data), and IL-6 (pg/mL) (baseline data) as mediator. The same steps were followed for the mediation analysis.

## 3 Results

### 3.1 Included data

The main analysis included a total of 1927 participants with complete data.

Missing variables affected BMI data (n = 12, 0.6 %), APOE ε4 status (n = 7, 0.4 %), and creatinine/eGFR (n = 19, 0.9 %). They were therefore imputed.

Plasma Aß_40_ (pg/mL), amyloid ratio (Aß_42_/Aß_40_), Tau, p-Tau_181_ (pg/mL), NfL (pg/mL), plasma Glucose (mg/dL), HbA1c (%), and IL-6 (pg/mL) levels were strongly right skewed, therefore, log-transformed for the linear regression and mediation analyses. Plasma Aß_42_ (pg/mL) and total Hippocampus volume (mm^3^) had a normal distribution and were kept in their raw form (otherwise distorted by log-transformation). MMSE and TMT-B total scores were converted to categorical data based on relevant cutoff values (24 points for MMSE and 90 s for TMT-B).

### 3.2 Study population

#### 3.2.1 Characteristics of the study population

Around one-quarter of the included cases were diagnosed with T_2_DM (24.13 %). The median age was 66 years, and 62 % were females. For age and sex, no statistically significant differences were observed between cases with and without T_2_DM. Details on the total population and group comparison are provided in Table 1.

**Table 1:** Characteristics of study participants and Type 2 diabetes-related comparison.

**Demographical characteristics**	All cases		Type 2 Diabetes Mellitus		
	**Complete**	**Overall** N = 1927 (100 %)^1^	**No** n = 1462 (75,87 %)^1^	**Yes** n = 465 (24,13 %)^1^	**p-value**^2^
Age (years)	1927	66 (60, 72)	66 (59, 72)	66 (61, 72)	0.8
Sex (Female)	1927	1201 (62 %)	922 (63 %)	276 (60 %)	0.2
Ethnicity	1927				**<0.001**
White		860 (45 %)	752 (51 %)	108 (23 %)	
Hispanic		834 (43 %)	532 (36 %)	302 (65 %)	
Blac		233 (12 %)	178 (12 %)	55 (12 %)	
Retire	1918	1081 (56 %)	812 (56 %)	269 (58 %)	0.3
Missing value		9	5	4	
Education (years)	1927	14.0 (11.0, 16.0)	14.0 (12.0, 16.0)	12.0 (7.0, 15.0)	**<0.001**
**Biological characteristics**					
APOE ε4 positivity	1927	492 (26 %)	396 (27 %)	96 (21 %)	**0.006**
BMI	1927	29 (26, 33)	29 (25, 33)	31 (28, 35)	**<0.001**
eGFR (mL/min/1.73 m^2^)	1927	87 (72, 97)	86 (72, 96)	91 (74, 99)	**0.003**
Glucose (mg/mL)	1908	98 (90, 111)	94 (88, 101)	136 (112, 172)	**<0.001**
Missing value		19	12	7	
HbA1c (%)	1902	5.60 (5.30, 6.10)	5.40 (5.20, 5.70)	7.10 (6.50, 8.43)	**<0.001**
Missing value		25	16	9	
Interleukin-6 levels (pg/mL)	1927	0.94 (0.63, 1.39)	0.88 (0.60, 1.28)	1.15 (0.76, 1.72)	**<0.001**
**Behavioral characteristics**					
Alcohol consumption	1927	16 (0.8 %)	10 (0.7 %)	6 (1.3 %)	0.2
Tobacco smoking (current)	1927	112 (5.8 %)	75 (5.1 %)	37 (8.0 %)	**0.023**
**Comorbidities**					
Depression	1927	653 (34 %)	462 (32 %)	191 (41 %)	**<0.001**
Anxiety	1927	334 (17 %)	238 (16 %)	96 (21 %)	**0.030**
Hypertension	1927	1232 (64 %)	863 (59 %)	369 (79 %)	**<0.001**
Dyslipidemia	1927	1352 (70 %)	986 (67 %)	366 (79 %)	**<0.001**
Cardiovascular Disease	1927	138 (7.2 %)	96 (6.6 %)	42 (9.0 %)	0.072
Obesity	1927	883 (46 %)	616 (42 %)	267 (57 %)	**<0.001**
Chronic Kidney Disease	1927	72 (3.7 %)	43 (2.9 %)	29 (6.2 %)	**0.001**
**Cognitive characteristics**					
Cognitive impairment∗	1927	442 (23 %)	311 (21 %)	131 (28 %)	**0.002**
Dementia	1927	108 (5.6 %)	70 (4.8 %)	38 (8.2 %)	**0.006**
MMSE total score (points)	1924	29.00 (27.00, 30.00)	29.00 (27.00, 30.00)	28.00 (26.00, 29.00)	**<0.001**
Missing value		3	2	1	
Trail-Making-Test B Time(sec)	1899	90 (66, 145)	84 (63, 127)	112 (79, 237)	**<0.001**
Missing value		28	23	5	
Plasma Amyloid β_40_ (pg/mL)	1927	206 (175, 242)	202 (173, 235)	219 (181, 267)	**<0.001**
Plasma Amyloid β_42_ (pg/mL)	1927	10.25 (8.69, 11.90)	10.20 (8.61, 11.70)	10.60 (8.92, 12.50)	**<0.001**
Aß_42_/Aß_40_ Ratio	1927	0.050 (0.044, 0.056)	0.050 (0.044, 0.057)	0.048 (0.043, 0.054)	**<0.001**
Plasma Total Tau (pg/mL)	1927	2.07 (1.64, 2.60)	2.03 (1.63, 2.54)	2.20 (1.73, 2.74)	**<0.001**
Plasma p-Tau_181_ (pg/mL)	1927	1.74 (1.34, 2.46)	1.74 (1.35, 2.45)	1.73 (1.30, 2.47)	>0.9
Plasma NfL (pg/mL)	1927	13 (9, 20)	13 (9, 18)	16 (11, 24)	**<0.001**
Hippocampus total volume (mm^3^)	1927	6389 (5,866, 6908)	6419 (5,905, 6934)	6255 (5,737, 6810)	**0.001**
MRI Scanner	1927				0.2
Skyra		1479 (77 %)	1111 (76 %)	368 (79 %)	
Vida 1		448 (23 %)	351 (24 %)	97 (21 %)	

^1^ Median (IQR); n (%), ^2^ Wilcoxon rank sum test; Pearson's Chi-squared test; Fisher's exact test.

∗**Cognitive impairment:** MCI + Dementia.

**Aß:** Amyloid Beta, **APOE:** Apolipoprotein, **BMI:** Body-Mass Index, **HbA1c:** Glycated Hemoglobin A1c, **eGFR:** Estimated Glomerular Filtration Rate, **MMSE:** Mini-Mental-Status Examination, **MRI:** Magnetic Resonance Imaging, **NfL:** Neurofilament Light Chain, **p-Tau_181_:** Phosphorylated Tau 181.

T_2_DM was significantly more prevalent among Hispanics (65 % vs. 23 % White and 12 % Black), while more White participants did not have T_2_DM (51 % vs. 36 % Hispanic and 12 % Black, *p*-value<0.001). Participants with T_2_DM tend to be, on average, two years less educated than those without diabetes (12 vs. 14 years, *p*-value<0.001). Biologically, participants with T_2_DM had higher plasma levels of fasting Glucose, HbA1c, and IL-6 in addition to higher BMI. While the rate of alcohol consumption did not significantly differ between groups, participants with T_2_DM disclosed significantly higher Tobacco smoking (8 % vs. 5.1 %, *p*-value = 0.023).

Depression (41 % vs. 32 %, *p-*value<0.001), anxiety (21 % vs. 16 %, *p-*value = 0.030), hypertension (79 % vs. 59 %, *p*-value<0.001), obesity (57 % vs. 42 %, *p-*value<0.001), and dyslipidemia (79 % vs. 67 %, *p-*value<0.001) were significantly more prevalent in T_2_DM participants. All cognitive biomarkers, except plasma p-Tau_181_ levels, were significantly worse in the T_2_DM group (all *p*-values<0.001, except p-Tau_181_ not significant).

Remarkably, a lower number of participants with APOE ε4 alleles had T_2_DM (21 % vs. 27 %, *p-*value = 0.006). Similarly, although a higher number of participants with T_2_DM disclosed having chronic kidney dysfunction (6.2 % vs. 2.9 %, *p*-value = 0.001), eGFR was significantly higher in this T_2_DM group (91 vs. 86 mL/min/1.73 m^2^, *p*-value = 0.003).

#### 3.2.2 Diabetes characteristics and group comparisons

Of the 465 total cases with diabetes at baseline, most of them (n = 399 to 416) reported details on diabetes-related habits. The majority of participants with diabetes are under any type of treatment (88 %), following healthy eating habits (83 %), and keeping regular physical activity (64 %). Hispanic and Black participants disclosed younger ages of T_2_DM onset than White participants (50 vs. 59 years in White, *p*-value<0.001). Cognition and ethnicity-based group comparison are detailed in Table 2.

**Table 2:** Differences in Type 2 Diabetes-related information between cognitive and ethnic groups.

Characteristics	Total		Cognitive impairment (MCI + Dementia)			Only dementia			Ethnicity			
	Complete	Overall N = 465^a^	No N = 334^a^	Yes N = 131^a^	p-value^b^	No N = 427^a^	Yes N = 38^a^	p-value^c^	White N = 108^a^	Hispanic N = 302^a^	Black N = 55^a^	p-value^d^
**Age of onset (years)**	414	52 (45, 60)	52 (45, 60)	55 (45, 61)	0.6	52 (45, 60)	50 (41, 63)	0.4	59 (50, 67)	50 (45, 60)	50 (45, 56)	**<0.001**
Missing value		51	36	15		47	4		11	33	7	
**On treatment (any)**	416	368 (88 %)	263 (88 %)	105 (89 %)	0.8	338 (89 %)	30 (86 %)	0.6	85 (87 %)	239 (89 %)	44 (90 %)	0.8
Missing value		49	36	13		46	3		10	33	6	
**Treatment: Oral medication**	399	358 (90 %)	258 (91 %)	100 (87 %)	0.2	330 (90 %)	28 (82 %)	0.14	83 (88 %)	231 (89 %)	44 (96 %)	0.4
Missing value		66	50	16		62	4		14	43	9	
**Treatment: Insulin**	399	110 (28 %)	79 (28 %)	31 (27 %)	0.9	101 (28 %)	9 (26 %)	0.9	21 (22 %)	79 (31 %)	10 (22 %)	0.2
Missing value		66	50	16		62	4		14	43	9	
**Treatment: Diet**	399	228 (57 %)	164 (58 %)	64 (56 %)	0.7	209 (57 %)	19 (56 %)	0.9	54 (57 %)	145 (56 %)	29 (63 %)	0.7
Missing value		66	50	16		62	4		14	43	9	
**Other Treatment**	398	79 (20 %)	56 (20 %)	23 (20 %)	>0.9	73 (20 %)	6 (18 %)	0.7	22 (23 %)	43 (17 %)	14 (31 %)	**0.049**
Missing value		67	50	17		63	4		14	43	10	
**Healthy eating Habits**	415	344 (83 %)	246 (83 %)	98 (83 %)	>0.9	317 (83 %)	27 (77 %)	0.3	81 (84 %)	218 (81 %)	45 (92 %)	0.2
Missing value		50	37	13		47	3		11	33	6	
**Exercising**	415	267 (64 %)	194 (65 %)	73 (62 %)	0.5	244 (64 %)	23 (66 %)	0.9	66 (68 %)	165 (61 %)	36 (73 %)	0.2
Missing value		50	37	13		47	3		11	33	6	

a Median (IQR); n (%).

b Wilcoxon rank sum test; Pearson's Chi-squared test.

c Wilcoxon rank sum test; Fisher's exact test; Pearson's Chi-squared test.

d Kruskal-Wallis rank sum test; Pearson's Chi-squared test; Fisher's exact test.

#### 3.2.3 IL-6 levels and associations with metabolic biomarkers

**Figure 1:**
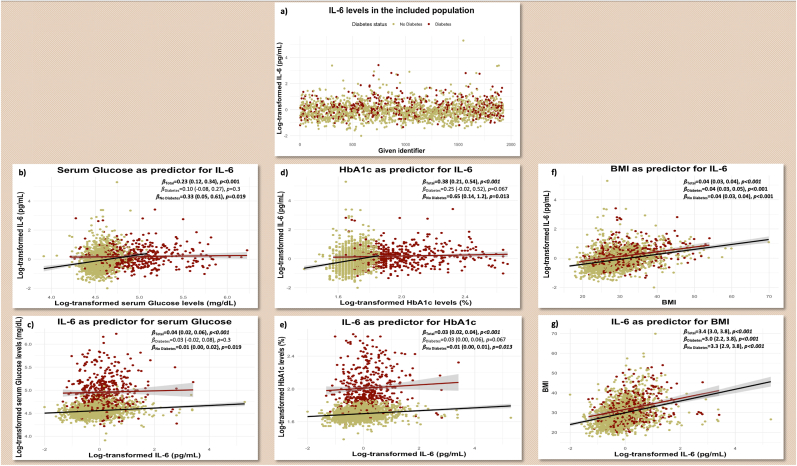
**Interleukin-6 levels in the study population and associations with different metabolic biomarkers. 1.a)** Interleukin-6 (IL-6) in the study population. **1.b) & 1.c)** Serum Glucose levels. **1.d) & 1.e)** Glycated Hemoglobin (HbA1c). **1.f) & 1.g)** Body Mass Index (BMI). **Footnote:** Interactions between Interleukin-6 and diabetes are visualized.

Higher IL-6 levels were significantly associated with higher Glucose (log-scaled adj. *β* = 0.04 [0.02, 0.06], *p-*value<0.001), and HbA1c (log-scaled adj. *β* = 0.03 [0.02, 0.04], *p-*value<0.001) values. Similarly, higher glucose (log-scaled adj. *β* = 0.23 [0.12, 0.34], *p-*value<0.001), and HbA1c (log-scaled adj. *β* = 0.038 [0.21, 0.54], *p-*value<0.001) levels were associated with increased IL-6 levels. Introducing an interaction term with diabetes showed significant effects. Interaction-specific visualization and stratification showed a dissociation in results, and the previous associations remained statistically significant only in non-T_2_DM participants. In T_2_DM, the associations lost their statistical significance. Results of the regression analysis are detailed and visualized in Fig. 1.

The bilateral association between higher IL-6 levels and higher BMI remained statistically significant with the same coefficients in both diagnostic groups (Fig. 1f & g).

#### 3.2.4 IL-6 and cognitive biomarkers

An increase of one unit log-transformed IL-6 levels was significantly associated with 35 % higher odds of cognitive impairment (OR = 1.35 [1.13, 1.60], *p*_*FDR*_*-*value<0.001) and 29 % higher odds of TMT-B ≥ 90 s (OR = 1.29 [1.07, 1.56], *p*_*FDR*_*-*value = 0.011). It was also significantly associated with a decrease in the total Hippocampus volume (adj. *ß* = −92 [−140, −44], *p*_*FDR*_*-*value<0.001) and Aß_42_/Aß_40_ ratio (log-scaled adj. *ß* = −0.02 [−0.04, 0.00], *p*_*FDR*_*-*value = 0.036), and an increase in the level of Aß_40_ (log-scaled adj. *β* = 0.04 [0.02, 0.06], *p*_*FDR*_*-*value<0.001), Aß_42_ (adj. *β* = 0.26 [0.08, 0.44], *p-*value = 0.007), total tau (log-scaled adj. *β* = 0.05 [0.02, 0.07], *p*_*FDR*_*-*value<0.001), p-Tau_181_ (log-scaled adj. *β* = 0.03 [0.00, 0.06], *p*_*FDR*_*-*value = 0.048), and NfL (log-scaled adj. *β* = 0.10 [0.07, 0.14], *p*_*FDR*_*-*value<0.001). No significant association was observed between IL-6 and the odds of MMSE ≤24 points (Table 3).

**Table 3:** Association between Type 2 Diabetes Mellitus, Interleukin-6, and biomarkers of cognitive function.

Dependent variables	T_2_DM as independent variable						IL-6 as independent variable			
	N	Event	β^1^/OR^2^	95 % CI	p-value	p_FDR_-value	β^1^/OR^2^	95 % CI	p-value	p_FDR_-value
Cognitive Impairment^a^	1927	442	1.12 c	0.87, 1.45	0.400	0.400	1.35 c	1.13, 1.60	**<0.001**	**<0.001**
MMSE total score ≤24 points	1924	223	0.77 c	0.53, 1.11	0.200	0.222	1.08 c	0.82, 1.41	0.600	0.600
Trail Making Test-B Time ≥90 s	1899	972	1.44 c	1.09, 1.90	**0.009**	**0.020**	1.29 c	1.07, 1.56	**0.008**	**0.011**
Hippocampus total volume (mm^3^)	1927	–	−89 b^,^^d^	−161, −17	**0.016**	**0.023**	−92 b^,^^d^	−140, −44	**<0.001**	**<0.001**
Log-transformed plasma Aβ40 (pg/mL)	1927	–	0.09 b^,^^e^	0.07, 0.12	**<0.001**	**<0.001**	0.04 b^,^^e^	0.02, 0.06	**<0.001**	**<0.001**
Plasma Aβ42 (pg/mL)	1927	–	0.63 b^,^^e^	0.36, 0.89	**<0.001**	**<0.001**	0.26 b^,^^e^	0.08, 0.44	**0.004**	**0.007**
Log-transformed Aβ42/Aβ40 Ratio	1927	–	−0.04 b^,^^e^	−0.06, −0.01	**0.010**	**0.020**	−0.02 b^,^^e^	−0.04, 0.00	**0.029**	**0.036**
Log-transformed plasma Total Tau (pg/mL)	1927	–	0.05 b^,^^e^	0.01, 0.09	**0.012**	**0.020**	0.05 b^,^^e^	0.02, 0.07	**<0.001**	**<0.001**
Log-transformed plasma p-Tau-181 (pg/mL)	1927	–	0.04 b^,^^e^	−0.01, 0.09	0.091	0.114	0.03 b^,^^e^	0.00, 0.06	**0.043**	**0.048**
Log-transformed plasma NfL (pg/mL)	1927	–	0.24 b^,^^e^	0.18, 0.29	**<0.001**	**<0.001**	0.10 b^,^^e^	0.07, 0.14	**<0.001**	**<0.001**
Log-transformed IL-6 (pg/mL)	1927	–	0.12	0.05, 0.19	**<0.001**	–	–	–	–	–

**Aß:** Amyloid Beta, **CI:** Confidence Interval, **IL-6:** Interleukin-6, **eGFR:** Estimated Glomerular Filtration Rate, **MMSE:** Mini-Mental Status Examination, **NfL:** Neurofilament Light Chain, **OR:** Odds Ratio, **p-Tau:** Phosphorylated Tau, **T_2_DM:** Type-2 Diabetes Mellitus.

a **Cognitive impairment:** MCI + Dementia.

b Multivariable linear regression model.

c Multivariable logistic regression modelModels adjusted for: Age (years) + Sex (Male, Female) + Ethnicity (White, Hispanic, Black) + APOE ε4 positivity (no allele or at least one allele) + Education (years) + Body mass index + Alcohol consumption + Current smoking.

d Hippocampus Model adjusted for: Age (years) + Sex (Male, Female) + Ethnicity (White, Hispanic, Black) + APOE ε4 positivity (no allele or at least one allele) + Education (years) + Body mass index + Alcohol consumption + Current smoking + **Intracranial volume (mm^3^) + Scanner**.

e ATN Models adjusted for: Age (years) + Sex (Male, Female) + Ethnicity (White, Hispanic, Black) + APOE ε4 positivity (no allele or at least one allele) + Education (years) + Body mass index + Alcohol consumption + Current smoking + **eGFR** (mL/min/1.73 m^2^).

**Figure 2:**
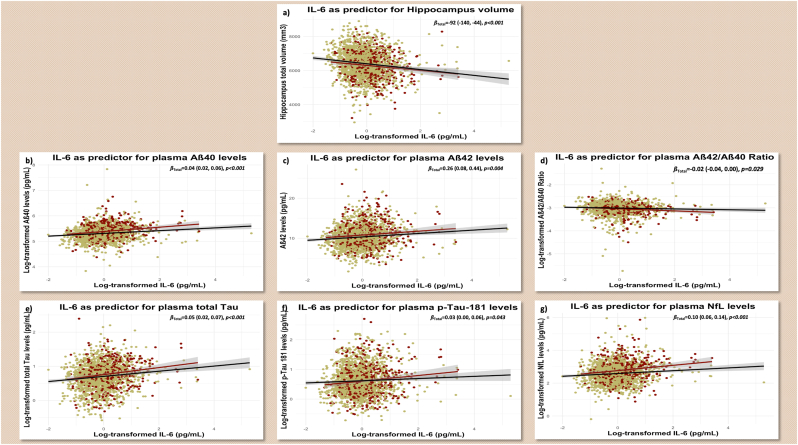
**Association between Interleukin-6 levels and different cognitive biomarkers.** Interactions between Interleukin-6 and diabetes are visualized. **2.a)** Hippocampus volume. **2.b)** Amyloid β_40_. **2.c)** Amyloid β_42_. **3.d)** Amyloid β_42_/_40_ ratio. **4.e)** Total Tau. **2.f)** Phosphorylated (p-)Tau_181_. **3.g)** Neurofilament Chain. **Footnote**: Amyloid, Tau, and Neurofilament models are adjusted for renal function. Hippocampus models are adjusted for intracranial volume and scanner.

No dissociation has been observed between T_2_DM and non-T_2_DM groups in this analysis (Fig. 2).

### 3.3 Diabetes and cognitive biomarkers

Diabetes at baseline (independently from the duration of the disease) was significantly associated with 44 % higher odds of TMT-B ≥ 90 s (OR = 1.44 [1.09, 1.90], *p*_*FDR*_*-*value = 0.009), a decrease in total Hippocampus volume (adj. *ß* = −89 [−161, −17], *p*_*FDR*_*-*value = 0.016) and Aß_42_/aß_40_ ratio (log-scaled adj. *ß* = −0.04 [−0.06, −0.01], *p*_*FDR*_*-*value = 0.010), and an increase of Aß_40_ (log-scaled adj. *β* = 0.09 [0.07, 0.12], *p*_*FDR*_*-*value<0.001), Aß_42_ (adj. *β* = 0.63 [0.36, 0.89], *p*_*FDR*_*-*value<0.001), total Tau (log-scaled adj. *β* = 0.05 [0.01, 0.09], *p*_*FDR*_*-*value = 0.012), and NfL levels (log-scaled adj. *β* = 0.24 [0.18, 0.29], *p*_*FDR*_*-*value<0.001).

There was also a significant association between having T_2_DM and higher IL-6 levels (log-scaled adj. *β* = 0.12 [0.05, 0.19], *p-*value<0.001).

There was no significant association between T_2_DM and the odds of cognitive impairment, MMSE total score ≤24 points, or p-Tau_181_ levels (Table 3).

Adding the interaction terms between ethnicity, T_2_DM, and IL-6 showed neither consistent nor significant results across different outcomes.

### 3.4 Mediation analysis: cross-sectional design

**Figure 3:**
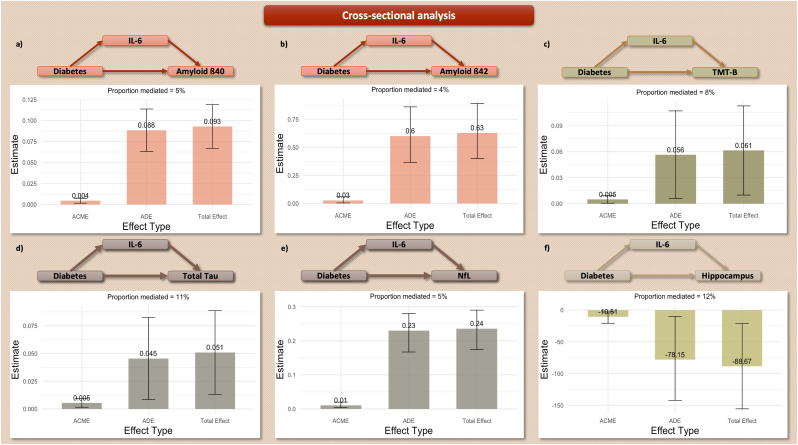
**Mediation analysis – Cross-sectional design.** Only statistically significant results are visualized. **3.a)** Amyloid β_40_. **3.b)** Amyloid β_42_. **3.c)** Trails-Making-Test B. **4.d)** Total Tau. **3.e)** Neurofilament Chain. **3.f)** Hippocampus volume. **Footnote**: ACME: Mediated Effect, ADE: Direct Effect. Amyloid, Tau, and Neurofilament models are adjusted for renal function. Hippocampus models are adjusted for intracranial volume and scanner.

The association between diabetes and biomarkers of cognitive impairment was significantly mediated by IL-6 (5 % for Aß_40_ ([1.5 %, 10 %], *p-*value<2 × 10^−16^), 4 % for Aß_42_ ([0.7 %, 11 %], *p-*value = 0.014), 8 % for pathological TMT-B ([0.2 %, 35 %], *p-*value = 0.046), 11 % for total Tau ([2.5 %, 40 %], *p-*value = 0.010), 5 % for NfL ([1.6 %, 8 %], *p-*value<2 × 10^−16^), and 12 % for Hippocampus atrophy ([3 %, 49 %], *p-*value = 0.004)). IL-6 did not show a significant mediating role in the association with the Aß_42_/Aß_40_ ratio (p-Tau_181_ was not included in the mediation analysis since it did not survive the FDR-adjusted associations). Statistically significant results are shown in Fig. 3.

### 3.5 Mediation analysis: retrospective longitudinal design

Particularities of this analyzed subgroup are analyzed and summarized in Supplementary Tables 1 and 2

**Figure 4:**
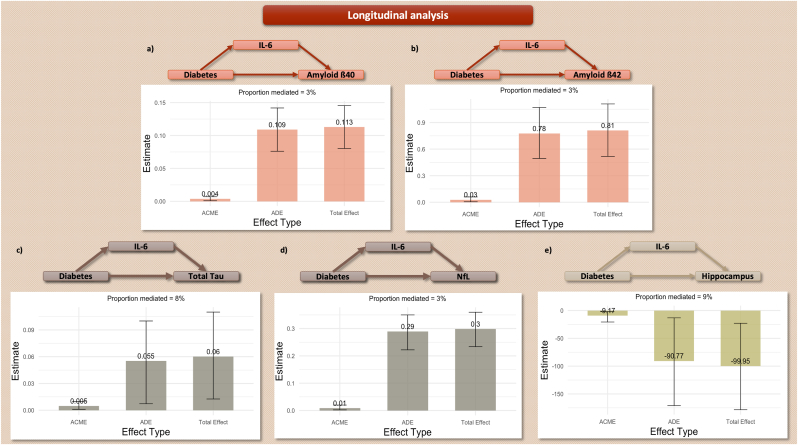
**Mediation analysis – Longitudinal design.** Only statistically significant results are visualized. **4.a)** Amyloid β_40_. **4.b)** Amyloid β_42_. **4.c)** Total Tau. **4.d)** Neurofilament Chain. **4.e)** Hippocampus volume. **Footnote**: ACME: Mediated Effect, ADE: Direct Effect. Amyloid, Tau, and Neurofilament models are adjusted for renal function. Hippocampus models are adjusted for intracranial volume and scanner.

The association between long-lasting diabetes and biomarkers of cognitive impairment in cases without previously diagnosed cognitive impairment was significantly mediated by IL-6 (3 % for Aß_40_ ([0.8 %, 8 %], *p-*value = 0.008), 3 % for Aß_42_ ([0.6 %, 8 %], *p-*value = 0.014), 8 % for total Tau ([1.6 %, 41 %], *p-*value = 0.016), 3 % for NfL ([0.7 %, 6 %], *p-*value = 0.008), and 9 % for Hippocampus atrophy ([1.5 %, 37 %], *p-*value = 0.024)). Here also, IL-6 did not show a significant mediating role in the association with Aß_42_/Aß_40_ ratio (TMT-B and p-Tau_181_ were excluded from the mediation analysis). Statistically significant findings are visualized in Fig. 4.

## 4 Discussion

The study showed significant associations between T_2_DM and several biomarkers of cognitive impairment, but not global cognition. IL-6 levels were higher among T_2_DM participants and mediated most observed associations. The results of the cross-sectional analyses were replicated in longitudinal analyses, where preexisting long-lasting T_2_DM had a significant predictive value on most cognitive biomarkers at baseline (particularly Hippocampus volume, total Tau, and NfL), despite excluding those priorly diagnosed with cognitive impairment. Several unexpected findings were revealed by this study. First, the lower proportion of APOE ε4 holders and higher eGFR levels in the T_2_DM participants. Second, the earlier onset age of T_2_DM in ethnic minorities. Finally, the T_2_DM-specific dissociation in the results of the association between IL-6 and diabetes-specific biomarkers (plasma Glucose and HbA1c). Although non-primary, these findings can help understand the main results in their context.

### 4.1 Diabetes in older adults

A French multicenter study in older adults (11.1 % with diabetes) showed that more adiposity (32.5 vs. 11.2 %, *p-*value<0.001), hypertension (77.4 vs. 59.8 %, *p-*value<0.001), dyslipidemia (60.5 vs. 48.9 %, *p-*value = 0.002), and cardiovascular history (19.3 vs. 7.7 %, *p-*value<0.001) were found in those with diabetes. The cognitive MMSE test scores were significantly worse in cases with diabetes than in those without. These results are comparable to our cohort. However, the prevalence of T_2_DM and obesity were much higher in our US-based cohort.

As expected, more participants with chronic kidney disease were among those who had diabetes (cross-sectional and longitudinal analyses). The unexpected finding was, however, the higher eGFR value in the T_2_DM group compared to the healthy control. For the current analysis, the eGFR was calculated based on the race-free CKD-EPI_2021_ formula (Miller et al., 2021). The results were verified using the CKD-EPI_2009_ formula-based eGFR (non-showed data), and the findings were replicated. The discrepancy might be explained by the early-stage diabetes-related hyperfiltration, where affected persons exhibit supraphysiological higher renal filtration rates in order to hyper-compensate for nephron damage (Tonneijck et al., 2017). Glomerular hyperfiltration is also a biomarker of early kidney function impairment in pre-diabetes and pre-hypertension (Palatini, 2012) and predicts higher mortality risk (Moriconi et al., 2023). In the longitudinal subset of the study, where only long-lasting diabetes was considered, although higher rates of chronic kidney disease were found in the T_2_DM group, no significant difference in eGFR values between the groups was observed. Other hypotheses might include the protective effect of medications (Tonneijck et al., 2017), eating habits (Kellar and Craft, 2020), and selection bias. This bias might be induced by the inclusion of more health-seeking and compliant participants in the diabetes groups, in addition to the low number of those with chronic kidney disease (only those at very early stages). This theory might be validated by the restrictiveness of the inclusion criteria, as participants with severe chronic kidney disease were not eligible for HABS-HD. This potential bias is, however, a strength in the estimation of the plasma ATN levels, since their levels might be significantly influenced by the renal function (Lehmann et al., 2023). All our plasma-based ATN-specific regression models (including mediation analysis) were adjusted for eGFR, which had a significant effect on the corresponding models, but did not change the significance level of the observed results.

Like in our current cohort, the French study reported fewer APOE ε4 carriers in the diabetes group than in controls (24.6 vs. 30.6 %, *p-*value = 0.05) (Frison et al., 2021). Previous studies found, however, an increased risk of insulin resistance in APOE (ε4/ε4) persons, exercising a synergetic interaction on the alteration of brain-blood barrier integrity (Padovani et al., 2025). A hypothetical explanation for these discrepancies might be associated with an earlier onset of AD, diabetes, and related complications in APOE ε4/ε4 persons, and, therefore, the lower likelihood of their participation in this cohort of community-dwelling adults. Furthermore, the mortality risk is higher in those with an earlier onset of T_2_DM (Life expectancy associated with different, 2023; Lin et al., 2024). Ethnic minorities, whose age of onset of T_2_DM was earlier than White counterparts, need particular attention, as suggested by our data.

### 4.2 Diabetes and cognition

Cross-sectional studies using UK-Biobank data showed significant associations between diabetes and specific cognitive domains (intelligence, numeric memory, reaction time …), while only one longitudinal study found a significant association between (pre-)diabetes and increased risk of cognitive decline (Fanelli et al., 2022). The French cohort study found that neurodegeneration, but not small vessel disease or AD pathology, mediated the association between diabetes and worse cognition (Frison et al., 2021). The authors evaluated neurodegeneration through the mean right and left cortical thickness, brain parenchymal fraction, mean glucose uptake at positron emission tomography (PET) scans, and total Hippocampus volume. In contrast, our current analysis considered the Hippocampus volume as an outcome, not a mediator. Hippocampus volume is a sensitive biomarker for early neurodegenerative processes and precedes other structures in predicting cognitive decline (Therriault et al., 2022). Furthermore, the French cohort found no mediating effect of the Aß_42_/Aß_40_ ratio, total Tau, and p-Tau between diabetes and cognitive decline (Frison et al., 2021). While they used cerebrospinal fluid (CSF)-based values, our current study included plasma-based measurements. CSF levels might be more sensitive than plasma levels. However, our choice is primarily due to data availability, and our analysis of plasma ATN levels was based on highly sensitive technologies enabling high accuracy of cognitive screenings in community-based settings (O'Bryant et al., 2022). In our study, T_2_DM predicted a significant decrease in the plasma-based Aß_42_/Aß_40_ ratio, reflecting an association with a sensitive biomarker of AD pathology. Similarly, our study found a significant association between T_2_DM and higher plasma total Tau and p-Tau_181_ levels, contradicting the French cohort findings, which found a significant association between diabetes and higher Amyloid load only at PET imaging (Frison et al., 2021). A recent meta-analysis showed, however, that pooled results of different studies, including the latter, are not in favor of a significant association between diabetes and Amyloid pathology, neither in PET nor in CSF measures (van Gils et al., 2024). The pooled data from PET- and CSF-Tau studies, in contrast, showed a significant association of Tau pathology with Glucose metabolism disorders and diabetes combined (van Gils et al., 2024).

A recent study in older patients with T_2_DM and overweight or obesity explored longitudinal associations between cognition and plasma levels of Aß_40_, Aß_42_, total Tau, p-Tau_181_, glial fibrillary acidic protein (GFAP), and NfL. Only the increase in GFAP and NfL levels over time was a significant predictor of incident cognitive impairment (Mielke et al., 2025). The selection bias related to the interventional intention of that study at baseline, the inclusion of high-risk participants with both T_2_DM and overweight and obesity, and the absence of a non-T_2_DM control group might explain the differences from our results. Furthermore, the included population was younger than 65 years at baseline, probably prior to the typical age of onset of detectable Amyloid pathology. Therefore, the study did not exclude the hypothesis of significant associations with Amyloid pathology in later ages (Mielke et al., 2025). The renal function had a significant impact on the predictive value of plasma-measured p-Tau_181_ (Mielke et al., 2025). In our study, plasma ATN models were adjusted for renal function, and their statistical significance was not affected after correcting for eGFR. This implies that higher plasma ATN levels in T_2_DM are not explained by a reduced renal clearance.

The association between insulin resistance and AD has been largely discussed. Insulin resistance precedes AD pathology and is involved in the impairment of Aβ clearance in the brain (Kellar and Craft, 2020). Plasma p-Tau is associated with Amyloid pathology and predicts cognitive decline (Gonzalez-Ortiz et al., 2024). The lack of significant associations in our study might indicate early stages of the AD continuum and needs to motivate further investigations (longitudinal analysis, comparison with the gold standard CSF levels …).

While the current study failed to find a significant association between T_2_DM and the overall state of cognitive impairment, another study showed that diabetes complications, mainly diabetic foot, microvascular, cerebrovascular, and cardiovascular diseases, were significant predictors of dementia onset within ten years of follow-up (Exalto et al., 2013). This might further suggest that our participants are at very early pre-clinical stages of diabetes-related complications.

Furthermore, insulin signaling disturbance in the brain is associated with impaired neurotransmitter signaling in preclinical models of psychosis (de Bartolomeis et al., 2023). This neurotransmitter hypothesis might be a further mechanism, but could not be explored by our cohort and needs further investigation.

### 4.3 Diabetes and IL-6

Higher IL-6 levels have a significant predictive value for incident diabetes. In a large, bi-ethnic US-based cohort, higher IL-6 predicted higher risks of incident diabetes and metabolic syndrome after 9.5 years of follow-up (Palermo et al., 2024). However, after stratification, the association was statistically significant only in non-Hispanic White participants, but not Black ones, despite higher IL-6 levels in the latter group at baseline (Palermo et al., 2024). This might be related to the ethnic discrepancy in the age of onset of diabetes and survival-related selection bias (Reeves et al., 2022). This age gap was also demonstrated in our study.

### 4.4 Cognition and IL-6

Several studies described the association between high IL-6 levels and cognitive impairment. A large UK-Biobank population study found a significant association between high IL-6 levels, cortical and subcortical atrophy (including the Hippocampus), and all-cause dementia in the longitudinal analysis (Zhao et al., 2024). The findings of the Berlin Aging Study showed a significant association between higher IL-6 levels and poorer executive function and processing speed in older adults (≥60 years) (Tegeler et al., 2016). In contrast, data from the Mayo Clinic Study of Aging did not show a significant association between IL-6 levels and domain-specific z-scores after adjusting for confounders. However, the latter study revealed a significant association between higher IL-6 levels and the concomitant odds of MCI in the cross-sectional analysis, but not incident cases during the follow-up (Wennberg et al., 2019). IL-6 levels exhibit both between and within individual variations in the long term, which might reduce the specificity and statistical power of prospective predictive models.

### 4.5 T_2_DM, cognition, and IL-6

In the UK-based Edinburgh Type 2 Diabetes Study, higher baseline levels of IL-6 were significantly associated with a decline in abstract reasoning and executive function within a 10-year follow-up period (Sluiman et al., 2022). IL-6 levels have not demonstrated a solid predictive value on global cognition (Sluiman et al., 2022), which confirms our current findings and highlights the domain-specific association between cognition, IL-6, and T_2_DM.

The difference between participants with and without T_2_DM in the statistical significance of association between higher IL-6 levels, and diabetes-related biomarkers (plasma Glucose and HbA1c) levels raises new questions on the eventual beneficial effect of diabetes-related therapy (medication, diet, regular physical activity) on down-regulating diabetes-related systemic inflammation. Previous interventional studies did not show a significant impact of insulin and metformin on the inflammatory biomarkers after a follow-up of 14 weeks, despite their positive effect in regulating blood glucose levels (Pradhan et al., 2009). This does not exclude, however, the long-term effects of various therapeutic interventions (different diabetes-regulating medications, mental health support, stress reduction (Rosenkranz et al., 2013), physical activity (Nishida et al., 2014), and Mediterranean diet (Frye et al., 2024)) on systemic inflammation in T_2_DM. These protective factors have also been associated with a decrease in dementia incidence (Mukadam et al., 2024).

### 4.6 Potential mechanisms

Previous in vitro and animal studies are in favor of a mediating role of IL-6 in the association between metabolic disorders and Alzheimer's pathology (Lyra et al., 2021; De Felice and Ferreira, 2014; Zhang et al., 2023). Systemic inflammation can reach the brain structures through an impaired blood-brain barrier (Elwood et al., 2017). The Vagus pathway is a further structure involved in the mediation between the cholinergic system, metabolic dysfunction, and inflammation (Pavlov and Tracey, 2012). Patients with AD have high IL-6 levels in the brain and plasma, and systemic IL-6 levels correlated with biomarkers of neuroinflammation and worse cognition (Lyra et al., 2021). On the brain level, there is a positive feedback effect between Aβ accumulation and activation of immunological cells, particularly astrocytes, known for their ability to produce IL-6 (Lyra et al., 2021). Similar inflammatory biomarkers were found in the hippocampus and hypothalamus of AD patients (Lyra et al., 2021), and the Hippocampus dysfunction in AD is comparable to hypothalamic dysregulation in metabolic disorders (De Felice and Ferreira, 2014). IL-6 receptors are largely expressed in the hippocampus of rodents; blocking circulating IL-6 prolonged long-term memory in freely moving rats (Balschun et al., 2004). Furthermore, neutralizing IL-6 in the brains of AD mice had a concomitant beneficial effect on their memory, peripheral glucose intolerance, and systemic IL-6 levels (Lyra et al., 2021).

### 4.7 Summary

To summarize, the study showed a significant association between T_2_DM, executive function (only cross-sectional), Hippocampus atrophy, plasma Amyloid, Tau, and NfL levels. IL-6 mediated significantly most of these associations. These results, in addition to the absence of association with clinically relevant global cognitive impairment, might translate initial stages of the T_2_DM-dementia pathology, where kidney function and Amyloid production are in hyper-compensatory stages, despite the significant concomitant decrease of the Aß_42_/Aß_40_ ratio. Furthermore, despite the significant association with IL-6, p-Tau_181_ missed the statistical levels of significance in the association with T_2_DM and needs to be monitored in the longitudinal course and eventually compared with corresponding CSF levels. The dissociations observed between T_2_DM and non-T_2_DM groups in the IL-6-based associative analyses might indicate a beneficial effect of mediation, healthy diet, and physical activity, as most participants with T_2_DM seem to be compliant with at least one of these protective factors, and this hypothesis needs to be explored by larger interventional and registry/population-based studies.

The mediating effect of IL-6 was modest despite its statistical significance. The findings are consistent with the complexity and multifactorial nature of inflammatory pathways. Cytokines contribute incrementally within inflammatory networks rather than having each a dominant effect (Kumari et al., 2024). Small changes in key mediators might have cumulative effects at a population level (Leng et al., 2023), particularly in vulnerable groups affected by chronic diseases such as T_2_DM. The current study focused only on IL-6, but other factors involved in the mediating path need to be further explored.

### 4.8 Strengths

This is the first study based on two frameworks (cross-sectional and longitudinal) to assess the association between diabetes and biomarkers of AD and the mediating role of IL-6. The large number of included participants from various ethnic backgrounds, the large set and completeness of the collected data, and the monocentricity and homogeneity of the assessment methods are further strengths of this study. The use of a non-parametric bootstrapping method to estimate 95 % CI in the mediation analysis presents a methodological novelty.

### 4.9 Limitations

The retrospective collection of medical history is the main limitation of the study. While it is less probable that there is a recall bias regarding the date of onset of diabetes, some participants, particularly those with cognitive impairment, might have provided imprecise dates. However, these cases were removed from the longitudinal analysis. Furthermore, diabetes might remain underdiagnosed for years, and many excluded participants from the longitudinal mediation analysis might have had diabetes, thus undiagnosed and first assessed during the recruitment in this cohort. This diagnosis bias might also have affected the assessment of cognitive impairment, as participants might have had a subtle form of cognitive impairment but remained undiagnosed until being recruited in the HABS-HD study.

While we acknowledge that self-reporting might induce some recall bias and misclassification, and the retrospective design of data collection can lead to incomplete information, potentially impacting the internal validity, several strategies were implemented to mitigate these risks. First, we validated self-reported diagnoses through additional structured and standardized questionnaires about the type of diabetes (type 1 was excluded), age of onset, detailed medication, and other interventions such as diet and physical activities. All participants underwent blood sampling, including HbA1c and fasting Glucose levels, to enhance diagnosis accuracy and detect new cases. We also compared in Table 1 the cases with and without T_2_DM and saw statistically significant differences in relevant variables. Second, we evaluated the comparability of our results with published studies (higher incidence of T_2_DM in the American population, earlier age of onset in ethnic minorities, significantly higher cardiometabolic risk profile …).

A further limitation is the potential selection bias arising from community-based recruitment and snowball sampling. This non-random sampling increases the risk of selection bias by including participants who are more socially connected and engaged within community networks. The aim of the source cohort was to include ethnic minorities rarely involved in clinical studies and difficult to reach in other settings (nursing homes, mental healthcare structures, clinical trials …). Ethnic minorities might exhibit reduced trust in medical research, and the snowball sampling allowed for advertising and encouraging their engagement in this study. The originality of our study is the focus on community-dwelling middle-aged and older adults, while equally representing different communities, in contrast with previous studies performed in clinical settings and centered around white communities. However, the current results might not apply to the total population or more severely affected persons.

Several confounders were not assessed in the source data. A healthy lifestyle, including a Mediterranean and fiber-rich diet, intermittent fasting, regular sleep habits, and accelerometer-measured regular exercise, for example, might play a protective role. We included BMI as a surrogate biomarker of a healthy lifestyle, but it is also important to acknowledge the limitations related to these unmeasured covariates. Another limitation is related to missing data on IL-6, Hippocampus volume, Amyloid, Tau, and NfL pathologies. Despite that, the number of recruited participants remained high compared to previous cohorts.

## 5 Conclusions

IL-6 mediates the association between long-lasting T_2_DM and AD biomarkers in middle-aged and older community-dwelling adults without previously diagnosed cognitive impairment. Despite the statistical significance of the observed associations, the mediating role of IL-6 does not explain the totality of the diabetes-related effect. This suggests other mechanisms besides IL-6-mediated neuroinflammation. More studies are needed to better understand further mediating risk factors, cluster patients based on associated predisposing factors, and orient toward personalized treatment.

## CRediT authorship contribution statement

**Asma Hallab:** Conceptualization, Data curation, Formal analysis, Investigation, Methodology, Project administration, Supervision, Validation, Visualization, Writing – original draft, Writing – review & editing. Members of the HABS-HD are listed as a group author to acknowledge the collection and provision of the data used in this study, as well as the funding acquisition.

## Ethical approval

All procedures contributing to this work comply with the ethical standards of the relevant national and institutional committees on human experimentation and with the Helsinki Declaration of 1975, as revised in 2013. Ethical approval was obtained from the local institutional review board (North Texas IRB Board). Participants gave written informed consent. The current research is based on a secondary analysis of anonymized data and was performed in compliance with the data use agreement (DUA with Hallab) and followed by an authorization for publication.

## Authorization for publication

The principal investigator and study director of the HABS-HD study revised the current version and ensured its compliance with DUA and authorized the publication of the manuscript. Vectors used in the graphical abstract are copyright-free (Brain downloaded from Freepik, and images AI-generated from OpenAI).

## Authorship

AH has full access to all of the data and takes responsibility for the integrity of the data and the accuracy of the analysis, visualization, drafting, and editing of the manuscript.

## Data availability

Data can be acquired by qualified researchers after an official request (asma.hallab@charite.de).

## Funding

“Research reported on this publication was supported by the National Institute on Aging of the National Institutes of Health under Award Numbers R01AG054073, R01AG058533, R01AG070862, P41EB015922, and U19AG078109. The content is solely the responsibility of the authors and does not necessarily represent the official views of the 10.13039/100000002National Institutes of Health.” AH did not receive any specific grant from funding agencies in the public, commercial, or not-for-profit sector for the generation of this work.

## Declaration of competing interest

None. The authors have nothing to declare.

## References

[bib1] Balschun D., Wetzel W., Del Rey A., Pitossi F., Schneider H., Zuschratter W. (2004). Interleukin-6: a cytokine to forget. Faseb j.

[bib2] de Bartolomeis A., De Simone G., De Prisco M., Barone A., Napoli R., Beguinot F. (2023). Insulin effects on core neurotransmitter pathways involved in schizophrenia neurobiology: a meta-analysis of preclinical studies. Implications for the treatment. Mol Psychiatry.

[bib3] Biessels G.J., Strachan M.W., Visseren F.L., Kappelle L.J., Whitmer R.A. (2014). Dementia and cognitive decline in type 2 diabetes and prediabetic stages: towards targeted interventions. Lancet Diabetes Endocrinol..

[bib4] Bowker N., Shah R.L., Sharp S.J., Luan Ja, Stewart I.D., Wheeler E. (2020). Meta-analysis investigating the role of interleukin-6 mediated inflammation in type 2 diabetes. EBioMedicine.

[bib5] Daniele G., Guardado Mendoza R., Winnier D., Fiorentino T.V., Pengou Z., Cornell J. (2014). The inflammatory status score including IL-6, TNF-α, osteopontin, fractalkine, MCP-1 and adiponectin underlies whole-body insulin resistance and hyperglycemia in type 2 diabetes mellitus. Acta Diabetol..

[bib6] Eid S., Sas K.M., Abcouwer S.F., Feldman E.L., Gardner T.W., Pennathur S. (2019). New insights into the mechanisms of diabetic complications: role of lipids and lipid metabolism. Diabetologia.

[bib7] von Elm E., Altman D.G., Egger M., Pocock S.J., Gøtzsche P.C., Vandenbroucke J.P. (2007). The Strengthening the Reporting of Observational Studies in Epidemiology (STROBE) statement: guidelines for reporting observational studies. Lancet.

[bib8] Elwood E., Lim Z., Naveed H., Galea I. (2017). The effect of systemic inflammation on human brain barrier function. Brain Behav. Immun..

[bib9] Exalto L.G., Biessels G.J., Karter A.J., Huang E.S., Katon W.J., Minkoff J.R. (2013). Risk score for prediction of 10 year dementia risk in individuals with type 2 diabetes: a cohort study. Lancet Diabetes Endocrinol..

[bib10] Fanelli G., Mota N.R., Salas-Salvadó J., Bulló M., Fernandez-Aranda F., Camacho-Barcia L. (2022). The link between cognition and somatic conditions related to insulin resistance in the UK Biobank study cohort: a systematic review. Neurosci. Biobehav. Rev..

[bib11] De Felice F.G., Ferreira S.T. (2014). Inflammation, defective insulin signaling, and mitochondrial dysfunction as common molecular denominators connecting type 2 diabetes to alzheimer disease. Diabetes.

[bib12] Feng S., Wang T., Su Y., Yan J., Wang Y., Zhang Z. (2025). Global burden, risk factors, and projections of early-onset dementia: insights from the Global Burden of Disease Study 2021. Ageing Res. Rev..

[bib13] Folstein M.F., Folstein S.E., McHugh P.R. (1975). "Mini-mental state". A practical method for grading the cognitive state of patients for the clinician. J. Psychiatr. Res..

[bib14] Frison E., Proust-Lima C., Mangin J.-F., Habert M.-O., Bombois S., Ousset P.-J. (2021). Diabetes mellitus and cognition. Neurology.

[bib15] Frye B.M., Negrey J.D., Johnson C.S.C., Kim J., Barcus R.A., Lockhart S.N. (2024). Mediterranean diet protects against a neuroinflammatory cortical transcriptome: associations with brain volumetrics, peripheral inflammation, social isolation, and anxiety in nonhuman primates (Macaca fascicularis). Brain Behav. Immun..

[bib16] Geijselaers S.L.C., Sep S.J.S., Stehouwer C.D.A., Biessels G.J. (2015). Glucose regulation, cognition, and brain MRI in type 2 diabetes: a systematic review. Lancet Diabetes Endocrinol..

[bib17] van Gils V., Rizzo M., Côté J., Viechtbauer W., Fanelli G., Salas-Salvadó J. (2024). The association of glucose metabolism measures and diabetes status with Alzheimer's disease biomarkers of amyloid and tau: a systematic review and meta-analysis. Neurosci. Biobehav. Rev..

[bib18] Gonzalez-Ortiz F., Kirsebom B.-E., Contador J., Tanley J.E., Selnes P., Gísladóttir B. (2024). Plasma brain-derived tau is an amyloid-associated neurodegeneration biomarker in Alzheimer's disease. Nat. Commun..

[bib19] Gunes A., Schmitt C., Bilodeau L., Huet C., Belblidia A., Baldwin C. (2023). IL-6 trans-signaling is increased in diabetes, impacted by glucolipotoxicity, and associated with liver stiffness and Fibrosis in fatty liver disease. Diabetes.

[bib20] Gunter J., Thostenson K., Borowski B., Reid R., Arani A., Bernstein M. (2017). ADNI-3 MRI protocol. Alzheimer's Dement..

[bib21] Heni M. (2024). The insulin resistant brain: impact on whole-body metabolism and body fat distribution. Diabetologia.

[bib22] Jørgensen I.F., Aguayo-Orozco A., Lademann M., Brunak S. (2020). Age-stratified longitudinal study of Alzheimer's and vascular dementia patients. Alzheimer's Dement..

[bib23] Kalaria R.N., Maestre G.E., Arizaga R., Friedland R.P., Galasko D., Hall K. (2008). Alzheimer's disease and vascular dementia in developing countries: prevalence, management, and risk factors. Lancet Neurol..

[bib24] Kellar D., Craft S. (2020). Brain insulin resistance in Alzheimer's disease and related disorders: mechanisms and therapeutic approaches. Lancet Neurol..

[bib25] Kim J., Yoon S., Lee S., Hong H., Ha E., Joo Y. (2020). A double-hit of stress and low-grade inflammation on functional brain network mediates posttraumatic stress symptoms. Nat. Commun..

[bib26] Kullmann S., Valenta V., Wagner R., Tschritter O., Machann J., Häring H.U. (2020). Brain insulin sensitivity is linked to adiposity and body fat distribution. Nat. Commun..

[bib27] Kumari S., Dhapola R., Sharma P., Nagar P., Medhi B., HariKrishnaReddy D. (2024). The impact of cytokines in neuroinflammation-mediated stroke. Cytokine Growth Factor Rev..

[bib28] Leclerc M., Bourassa P., Tremblay C., Caron V., Sugère C., Emond V. (2023). Cerebrovascular insulin receptors are defective in Alzheimer's disease. Brain.

[bib29] Lehmann S., Schraen-Maschke S., Vidal J-S, Delaby C., Blanc F., Paquet C. (2023). Plasma phosphorylated tau 181 predicts amyloid status and conversion to dementia stage dependent on renal function. Journal of Neurology, Neurosurgery &&; Psychiatry.

[bib30] Leng F., Hinz R., Gentleman S., Hampshire A., Dani M., Brooks D.J. (2023). Neuroinflammation is independently associated with brain network dysfunction in Alzheimer's disease. Mol. Psychiatr..

[bib31] (2023). Life expectancy associated with different ages at diagnosis of type 2 diabetes in high-income countries: 23 million person-years of observation. Lancet Diabetes Endocrinol..

[bib32] Lin B., Coleman R.L., Bragg F., Maddaloni E., Holman R.R., Adler A.I. (2024). Younger-onset compared with later-onset type 2 diabetes: an analysis of the UK Prospective Diabetes Study (UKPDS) with up to 30 years of follow-up (UKPDS 92). Lancet Diabetes Endocrinol..

[bib33] Lyra e Silva NM., Gonçalves R.A., Pascoal T.A., Lima-Filho R.A.S., Resende EdPF., Vieira E.L.M. (2021). Pro-inflammatory interleukin-6 signaling links cognitive impairments and peripheral metabolic alterations in Alzheimer's disease. Transl. Psychiatry.

[bib34] Mendenhall E., Kohrt B.A., Norris S.A., Ndetei D., Prabhakaran D. (2017). Non-communicable disease syndemics: poverty, depression, and diabetes among low-income populations. Lancet.

[bib35] Mielke M.M., Evans J.K., Neiberg R.H., Molina-Henry D.P., Marcovina S.M., Johnson K.C. (2025). Alzheimer disease blood biomarkers and cognition among individuals with diabetes and overweight or obesity. JAMA Netw. Open.

[bib36] Miller W.G., Kaufman H.W., Levey A.S., Straseski J.A., Wilhelms K.W., Yu H.Y. (2021). National kidney foundation laboratory engagement working group recommendations for implementing the CKD-EPI 2021 race-free equations for estimated glomerular filtration rate: practical guidance for clinical laboratories. Clin. Chem..

[bib37] Moriconi D., Sacchetta L., Chiriacò M., Nesti L., Forotti G., Natali A. (2023). Glomerular hyperfiltration predicts kidney function decline and mortality in type 1 and type 2 diabetes: a 21-year longitudinal study. Diabetes Care.

[bib38] Mukadam N., Wolters F.J., Walsh S., Wallace L., Brayne C., Matthews F.E. (2024). Changes in prevalence and incidence of dementia and risk factors for dementia: an analysis from cohort studies. Lancet Public Health.

[bib39] Nichols E., Steinmetz J.D., Vollset S.E., Fukutaki K., Chalek J., Abd-Allah F. (2022). Estimation of the global prevalence of dementia in 2019 and forecasted prevalence in 2050: an analysis for the Global Burden of Disease Study 2019. Lancet Public Health.

[bib40] Nishida Y., Higaki Y., Taguchi N., Hara M., Nakamura K., Nanri H. (2014). Objectively measured physical activity and inflammatory cytokine levels in middle-aged Japanese people. Prev. Med..

[bib41] O'Bryant S.E., Johnson L.A., Barber R.C., Braskie M.N., Christian B., Hall J.R. (2021). The Health & Aging Brain among Latino Elders (HABLE) study methods and participant characteristics. Alzheimer's Dement..

[bib42] O'Bryant S.E., Zhang F., Petersen M., Hall J.R., Johnson L.A., Yaffe K. (2022). A blood screening tool for detecting mild cognitive impairment and Alzheimer's disease among community-dwelling Mexican Americans and non-Hispanic Whites: a method for increasing representation of diverse populations in clinical research. Alzheimer's Dement..

[bib43] Osimo E.F., Baxter L.J., Lewis G., Jones P.B., Khandaker G.M. (2019). Prevalence of low-grade inflammation in depression: a systematic review and meta-analysis of CRP levels. Psychol. Med..

[bib44] Padovani A., Galli A., Bazzoli E., Tolassi C., Caratozzolo S., Gumina B. (2025). The role of insulin resistance and APOE genotype on blood-brain barrier integrity in Alzheimer's disease. Alzheimer's Dement..

[bib45] Palatini P. (2012). Glomerular hyperfiltration: a marker of early renal damage in pre-diabetes and pre-hypertension. Nephrol. Dial. Transplant..

[bib46] Palermo B.J., Wilkinson K.S., Plante T.B., Nicoli C.D., Judd S.E., Kamin Mukaz D. (2024). Interleukin-6, diabetes, and metabolic syndrome in a biracial cohort: the reasons for geographic and racial differences in stroke cohort. Diabetes Care.

[bib47] Palmer E.R., Morales-Muñoz I., Perry B.I., Marwaha S., Warwick E., Rogers J.C. (2024). Trajectories of inflammation in youth and risk of mental and cardiometabolic disorders in adulthood. JAMA Psychiatry.

[bib48] Pavlov V.A., Tracey K.J. (2012). The vagus nerve and the inflammatory reflex—linking immunity and metabolism. Nat. Rev. Endocrinol..

[bib49] Peters M.C., McGrath K.W., Hawkins G.A., Hastie A.T., Levy B.D., Israel E. (2016). Plasma interleukin-6 concentrations, metabolic dysfunction, and asthma severity: a cross-sectional analysis of two cohorts. Lancet Respir. Med..

[bib50] Pradhan A.D., Everett B.M., Cook N.R., Rifai N., Ridker P.M. (2009). Effects of initiating insulin and metformin on glycemic control and inflammatory biomarkers among patients with type 2 diabetes: the LANCET randomized trial. JAMA.

[bib51] Reeves A., Elliott M.R., Lewis T.T., Karvonen-Gutierrez C.A., Herman W.H., Harlow S.D. (2022). Study selection bias and racial or ethnic disparities in estimated age at onset of cardiometabolic disease among midlife women in the US. JAMA Netw. Open.

[bib52] Reitan R.M. (1958). Validity of the trail making test as an indicator of organic brain damage. Percept. Mot. Skills.

[bib53] Rosenkranz M.A., Davidson R.J., Maccoon D.G., Sheridan J.F., Kalin N.H., Lutz A. (2013). A comparison of mindfulness-based stress reduction and an active control in modulation of neurogenic inflammation. Brain Behav. Immun..

[bib54] Scherer T., Sakamoto K., Buettner C. (2021). Brain insulin signalling in metabolic homeostasis and disease. Nat. Rev. Endocrinol..

[bib55] van Sloten T.T., Sedaghat S., Carnethon M.R., Launer L.J., Stehouwer C.D.A. (2020). Cerebral microvascular complications of type 2 diabetes: stroke, cognitive dysfunction, and depression. Lancet Diabetes Endocrinol..

[bib56] van Sloten T.T., Luchsinger J.A., Launer L.J., Strachan M., Cukierman-Yaffe T., Gerstein H.C. (2024). Call for effective therapies for preventing dementia in people with type 2 diabetes. Lancet Diabetes Endocrinol..

[bib57] Sluiman A.J., McLachlan S., Forster R.B., Strachan M.W.J., Deary I.J., Price J.F. (2022). Higher baseline inflammatory marker levels predict greater cognitive decline in older people with type 2 diabetes: year 10 follow-up of the Edinburgh Type 2 Diabetes Study. Diabetologia.

[bib58] Tegeler C., O'Sullivan J.L., Bucholtz N., Goldeck D., Pawelec G., Steinhagen-Thiessen E. (2016). The inflammatory markers CRP, IL-6, and IL-10 are associated with cognitive function--data from the Berlin Aging Study II. Neurobiol. Aging.

[bib59] Therriault J., Pascoal T.A., Lussier F.Z., Tissot C., Chamoun M., Bezgin G. (2022). Biomarker modeling of Alzheimer's disease using PET-based Braak staging. Nat Aging.

[bib60] Thyreau B., Sato K., Fukuda H., Taki Y. (2018). Segmentation of the hippocampus by transferring algorithmic knowledge for large cohort processing. Med. Image Anal..

[bib61] Tomic D., Shaw J.E., Magliano D.J. (2022). The burden and risks of emerging complications of diabetes mellitus. Nat. Rev. Endocrinol..

[bib62] Tonneijck L., Muskiet M.H., Smits M.M., van Bommel E.J., Heerspink H.J., van Raalte D.H. (2017). Glomerular hyperfiltration in diabetes: mechanisms, clinical significance, and treatment. J. Am. Soc. Nephrol..

[bib63] Wennberg A.M.V., Hagen C.E., Machulda M.M., Knopman D.S., Petersen R.C., Mielke M.M. (2019). The cross-sectional and longitudinal associations between IL-6, IL-10, and TNFα and cognitive outcomes in the Mayo clinic study of aging. J Gerontol A Biol Sci Med Sci.

[bib64] Wolters F.J., Ikram M.A. (2019). Epidemiology of vascular dementia. Arterioscler. Thromb. Vasc. Biol..

[bib65] Zhang W., Xiao D., Mao Q., Xia H. (2023). Role of neuroinflammation in neurodegeneration development. Signal Transduct. Targeted Ther..

[bib66] Zhao Z., Zhang J., Wu Y., Xie M., Tao S., Lv Q. (2024). Plasma IL-6 levels and their association with brain health and dementia risk: a population-based cohort study. Brain Behav. Immun..

[bib67] Zhou B., Rayner A.W., Gregg E.W., Sheffer K.E., Carrillo-Larco R.M., Bennett J.E. (2024). Worldwide trends in diabetes prevalence and treatment from 1990 to 2022: a pooled analysis of 1108 population-representative studies with 141 million participants. Lancet.

