# Supplementary material for: Mediating role of Interleukin-6 in the predictive association of diabetes with Hippocampus atrophy, Amyloid, Tau, and Neurofilament pathology at pre-clinical stages of diabetes-related cognitive impairment
Source: Brain Behav Immun Health. 2025 Jun 16;47:101031. doi: 10.1016/j.bbih.2025.101031 (PMC12216742; doi:10.1016/j.bbih.2025.101031)
Supplement: Multimedia component 1 [file mmc1.docx]

**Supp. Table 1:** Characteristics of study participants and Type 2 diabetes-related comparison – Data subset of the longitudinal analysis

|  | **All cases** | | **Type 2 Diabetes Mellitus** | | |
| --- | --- | --- | --- | --- | --- |
| **Demographical characteristics** | **Complete** | **Overall**  N = 1,791 (100%)^1^ | **No**  n = 1,454 (81.2%)^1^ | **Yes**  n = 337 (18.8%)^1^ | **p-value**^2^ |
| Age (years) | 1,791 | 66 (60, 72) | 66 (59, 72) | 67 (62, 73) | **0.045** |
| Sex (Female) | 1,791 | 1,125 (63%) | 916 (63%) | 209 (62%) | 0.7 |
| Ethnicity | 1,791 |  |  |  | **<0.001** |
| White |  | 823 (46%) | 747 (51%) | 76 (23%) |  |
| Hispanic |  | 751 (42%) | 530 (36%) | 221 (66%) |  |
| Black |  | 217 (12%) | 177 (12%) | 40 (12%) |  |
| Retired | 1,783 | 1,012 (57%) | 805 (56%) | 207 (62%) | **0.033** |
| Missing value |  | 8 | 5 | 3 |  |
| Education (years) | 1,791 | 14.0 (11.0, 16.0) | 14.0 (12.0, 16.0) | 12.0 (7.0, 15.0) | **<0.001** |
| **Biological characteristics** |  |  |  |  |  |
| APOE ε4 positivity | 1,791 | 462 (26%) | 391 (27%) | 71 (21%) | **0.029** |
| BMI | 1,791 | 29.2 (25.7, 33.2) | 28.9 (25.4, 32.7) | 30.6 (27.4, 34.6) | **<0.001** |
| eGFR (mL/min/1.73 m^2^) | 1,791 | 87 (72, 97) | 86 (72, 96) | 89 (70, 99) | 0.3 |
| Glucose (mg/mL) | 1,773 | 97 (90, 108) | 94 (88, 101) | 136 (109, 175) | **<0.001** |
| Missing value |  | 18 | 12 | 6 |  |
| HbA1c (%) | 1,767 | 5.50 (5.30, 5.90) | 5.40 (5.20, 5.70) | 7.30 (6.40, 8.70) | **<0.001** |
| Missing value |  | 24 | 16 | 8 |  |
| Interleukin-6 levels (pg/mL) | 1,791 | 0.92 (0.62, 1.35) | 0.88 (0.60, 1.28) | 1.12 (0.76, 1.67) | **<0.001** |
| **Behavioral characteristics** |  |  |  |  |  |
| Alcohol use disorder | 1,791 | 14 (0.8%) | 10 (0.7%) | 4 (1.2%) | 0.3 |
| Tobacco smoking | 1,791 | 96 (5.4%) | 75 (5.2%) | 21 (6.2%) | 0.4 |
| **Comorbidities** |  |  |  |  |  |
| Depression | 1,791 | 596 (33%) | 458 (31%) | 138 (41%) | **<0.001** |
| Anxiety | 1,791 | 304 (17%) | 237 (16%) | 67 (20%) | 0.11 |
| Hypertension | 1,791 | 1,130 (63%) | 858 (59%) | 272 (81%) | **<0.001** |
| Dyslipidemia | 1,791 | 1,254 (70%) | 978 (67%) | 276 (82%) | **<0.001** |
| Cardiovascular Disease | 1,791 | 128 (7.1%) | 95 (6.5%) | 33 (9.8%) | **0.036** |
| Obesity | 1,791 | 799 (45%) | 616 (42%) | 183 (54%) | **<0.001** |
| Chronic Kidney Disease | 1,791 | 68 (3.8%) | 43 (3.0%) | 25 (7.4%) | **<0.001** |
| **Cognitive characteristics** |  |  |  |  |  |
| Cognitive impairment* | 1,791 | 394 (22%) | 303 (21%) | 91 (27%) | **0.014** |
| Dementia | 1,791 | 92 (5.1%) | 63 (4.3%) | 29 (8.6%) | **0.001** |
| MMSE total score (points) | 1,789 | 29.00 (27.00, 30.00) | 29.00 (27.00, 30.00) | 28.00 (26.00, 29.00) | **<0.001** |
| Missing value |  | 2 | 1 | 1 |  |
| Trail-Making-Test B Time(sec) | 1,768 | 89 (65, 139) | 84 (63, 126) | 113 (79, 226) | **<0.001** |
| Missing value |  | 23 | 19 | 4 |  |
| Plasma Amyloid ß_40_ (pg/mL) | 1,791 | 206 (175, 242) | 202 (173, 235) | 225 (185, 275) | **<0.001** |
| Plasma Amyloid ß_42_ (pg/mL) | 1,791 | 10.30 (8.72, 11.95) | 10.20 (8.62, 11.70) | 10.87 (9.04, 12.95) | **<0.001** |
| Aß_42_/Aß_40_ Ratio | 1,791 | 0.050 (0.044, 0.057) | 0.050 (0.044, 0.057) | 0.048 (0.043, 0.054) | **<0.001** |
| Plasma Total Tau (pg/mL) | 1,791 | 2.06 (1.63, 2.59) | 2.03 (1.62, 2.53) | 2.23 (1.74, 2.79) | **<0.001** |
| Plasma p-Tau_181_ (pg/mL) | 1,791 | 1.75 (1.35, 2.48) | 1.73 (1.34, 2.45) | 1.77 (1.40, 2.69) | **0.050** |
| Plasma NfL (pg/mL) | 1,791 | 14 (9, 20) | 13 (9, 18) | 18 (12, 26) | **<0.001** |
| Hippocampus total volume (mm^3^) | 1,791 | 6,391 (5,866, 6,910) | 6,420 (5,910, 6,934) | 6,214 (5,674, 6,749) | **<0.001** |
| MRI Scanner | 1,791 |  |  |  | 0.2 |
| Skyra |  | 1,372 (77%) | 1,104 (76%) | 268 (80%) |  |
| Vida 1 |  | 419 (23%) | 350 (24%) | 69 (20%) |  |
| ^1^ Median (IQR); n (%), ^2^ Wilcoxon rank sum test; Pearson’s Chi-squared test; Fisher’s exact test | | | | | |
| ***Cognitive impairment:** MCI + Dementia | | | | | |
| **Aß:** Amyloid Beta, **APOE:** Apolipoprotein, **BMI:** Body-Mass Index, **HbA1c:** Glycated Hemoglobin A1c, **eGFR:** Estimated Glomerular Filtration Rate, **MMSE:** Mini-Mental-Status Examination, **MRI:** Magnetic Resonance Imaging, **NfL:** Neurofilament Light Chain, **p-Tau_181_:** Phosphorylated Tau 181 | | | | | |
