# Supplementary material for: Mediating role of Interleukin-6 in the predictive association of diabetes with Hippocampus atrophy, Amyloid, Tau, and Neurofilament pathology at pre-clinical stages of diabetes-related cognitive impairment
Source: Brain Behav Immun Health. 2025 Jun 16;47:101031. doi: 10.1016/j.bbih.2025.101031 (PMC12216742; doi:10.1016/j.bbih.2025.101031)
Supplement: Multimedia component 2 [file mmc2.docx]

**Supp. Table 2:** Association between Type 2 Diabetes Mellitus, Interleukin-6, and the biomarkers of cognitive function – Data subset for the longitudinal analysis

|  |  |  | **T_2_DM as independent variable** | | | | **IL-6 as independent variable** | | | |
| --- | --- | --- | --- | --- | --- | --- | --- | --- | --- | --- |
| **Dependent variables** | **N** | **Event** | ***ß*^1^ / OR^2^** | **95% *CI*** | ***p-*value** | ***p_FDR_-*value** | ***ß*^1^ / OR^2^** | ***95% CI*** | ***p*-value** | ***p_FDR_-*value** |
| Incident cognitive Impairment**^♦^** | 1,791 | 394 | 1.03**^2^** | 0.77, 1.38 | 0.8 | 0.800 | 1.42**^2^** | 1.18, 1.70 | **<0.001** | **<0.001** |
| MMSE total score ≤ 24 points | 1,791 | 193 | 0.68**^2^** | 0.44, 1.03 | 0.067 | 0.084 | 1.09**^2^** | 0.81, 1.45 | 0.600 | 0.600 |
| Trail Making Test-B Time ≥ 90 sec | 1,768 | 880 | 1.32**^2^** | 0.97, 1.80 | 0.083 | 0.092 | 1.40**^2^** | 1.15, 1.71 | **<0.001** | **<0.001** |
| Hippocampus total volume (mm^3^) | 1,791 | — | -100**^1, *^** | -181, -19 | **0.016** | **0.023** | -92**^1, *^** | -142, -43 | **<0.001** | **<0.001** |
| Log-transformed plasma Aß40 (pg/mL) | 1,791 | — | 0.11**^1, **^** | 0.08, 0.14 | **<0.001** | **<0.001** | 0.04**^1, **^** | 0.02, 0.06 | **<0.001** | **<0.001** |
| Plasma Aß42 (pg/mL) | 1,791 | — | 0.08**^1, **^** | 0.50, 1.1 | **<0.001** | **<0.001** | 0.30**^1, **^** | 0.11, 0.48 | **0.002** | **0.003** |
| Log-transformed Aß42 / Aß40 Ratio | 1,791 | — | -0.04**^1, **^** | -0.07, -0.01 | **0.012** | **0.020** | -0.02**^1, **^** | -0.04, 0.00 | 0.092 | 0.102 |
| Log-transformed plasma Total Tau (pg/mL) | 1,791 | — | 0.06**^1, **^** | 0.02, 0.10 | **0.009** | **0.018** | 0.05**^1, **^** | 0.02, 0.08 | **<0.001** | **<0.001** |
| Log-transformed plasma p-Tau-181 (pg/mL) | 1,791 | — | 0.07**^1, **^** | 0.02, 0.12 | **0.005** | **0.013** | 0.03**^1, **^** | 0.00, 0.06 | 0.051 | 0.064 |
| Log-transformed plasma NfL (pg/mL) | 1,791 | — | 0.30**^1, **^** | 0.24, 0.36 | **<0.001** | **<0.001** | 0.10**^1, **^** | 0.06, 0.13 | **<0.001** | **<0.001** |
| Log-transformed IL-6 (pg/mL) | 1,791 | — | 0.10**^1^** | 0.03, 0.18 | **0.007** |  | — | — | — | — |
| **Aß:** Amyloid beta, **CI:** Confidence Interval, **IL-6:** Interleukin-6, **eGFR:** Estimated Glomerular Filtration Rate, **MMSE:** Mini-Mental Status Examination, **OR:** Odds Ratio, **p-Tau:** Phosphorylated Tau, **T_2_DM:** Type 2 Diabetes Mellitus | | | | | | | | | | |
| **^♦^Cognitive impairment:** MCI + Dementia | | | | | | | | | | |
| ^1^ Multivariable linear regression model, ^2^ Multivariable logistic regression model  Models adjusted for: Age (years) + Sex (Male, Female) + Ethnicity (White, Hispanic, Black) + APOE ε4 positivity (no allele or at least one allele) + Education (years) + Body mass index + Alcohol consumption + Current smoking  *Hippocampus Model adjusted for: Age (years) + Sex (Male, Female) + Ethnicity (White, Hispanic, Black) + APOE ε4 positivity (no allele or at least one allele) + Education (years) + Body mass index + Alcohol consumption + Current smoking + **Intracranial volume (mm^3^) + Scanner**  **ATN Models adjusted for: Age (years) + Sex (Male, Female) + Ethnicity (White, Hispanic, Black) + APOE ε4 positivity (no allele or at least one allele) + Education (years) + Body mass index + Alcohol consumption + Current smoking + **eGFR** (mL/min/1.73 m^2^) | | | | | | | | | | |
